# Toward Enhancing the Thermoelectric Properties of Bi_2_Te_3_ and Sb_2_Te_3_ Alloys by Co-Evaporation of Bi_2_Te_3_:Bi and Sb_2_Te_3_:Te

**DOI:** 10.3390/nano15040299

**Published:** 2025-02-16

**Authors:** Bernardo S. Dores, Marino J. Maciel, José H. Correia, Eliana M. F. Vieira

**Affiliations:** 1 CMEMS—UMINHO, University of Minho, 4800-058 Guimarães, Portugal; pg50262@alunos.uminho.pt (B.S.D.); mmaciel@dei.uminho.pt (M.J.M.); higino.correia@dei.uminho.pt (J.H.C.); 2 LABBELS—Associate Laboratory, 4710-057 Braga/Guimarães, Portugal

**Keywords:** thermoelectric technology, telluride alloys, thermal co-evaporation, power factor enhancement

## Abstract

In this work, we developed nanostructured Bi_2_Te_3_ and Sb_2_Te_3_ thin films by thermal co-evaporation of their alloys with corresponding pure elements (Bi, Sb, and Te). The films were fabricated on borosilicate glass at different substrate temperatures and deposition rates. At 300 °C, enhanced thermoelectric performance was demonstrated for *n*-type Bi_2_Te_3_:Bi and *p*-type Sb_2_Te_3_:Te, with Seebeck coefficients of 195 µV K^−1^ and 178 μV K^−1^, along with electrical conductivities of 4.6 × 10^4^ (Ω m)^−1^ and 6.9 × 10^4^ (Ω m)^−1^, resulting in maximum power factor values of 1.75 mW K^−2^ m^−1^ and 2.19 mW K^−2^ m^−1^, respectively. These values are found to be higher than some reported works in the literature, highlighting the advantage of not introducing additional elements to the system (such as extra doping, which induces complexity to the system). The structural properties, film morphology, and chemical composition of the optimized films were investigated using X-ray diffraction (XRD) and scanning electron microscopy combined with energy-dispersive X-ray spectroscopy (SEM-EDS). The films were found to be polycrystalline with preferred (0 0 6) and (0 1 5) orientations for Bi_2_Te_3_ and Sb_2_Te_3_ films, respectively, and stable rhombohedral phases. Additionally, a ring-shaped *p-n* thermoelectric device for localized heating/cooling was developed and a temperature difference of ~7 °C between the hot and cold zones was obtained using 4.8 mA of current (*J* = 0.068 mA/mm^2^).

## 1. Introduction

Green technologies such as solar photovoltaics, wind turbines, biomass energy, and hydrogen systems are increasingly recognized as practical answers to the twin problems of environmental sustainability and energy consumption. In addition to these well-known technologies, thermoelectric (TE) technology is increasingly acknowledged as a practical answer for renewable energy uses, such as power generation, if a temperature difference is maintained between two terminals of a TE module, or providing localized heating/cooling of an object connected to one side of a TE module when supplied by a direct current (DC). Bismuth telluride (Bi_2_Te_3_) and antimony telluride (Sb_2_Te_3_) are typically *n*-type and *p*-type, respectively. These TEs have been the most employed materials in large-scale industries, namely in refrigerators, power generators, and thermal sensors, and are considered the archetypal TE materials for room temperature applications [1,2,3]. Specifically, localized heating systems fabricated in thin-film technology have attracted considerable attention in frontier areas of research, specifically in nanoscience for nanomaterials characterization [4], in microfluidics systems integration for healthcare applications [5], and to increase the efficiency of alkali vapor cells through the SERF regime for biomedical applications [6]. Microheating devices based on TE materials can provide rapid response, temperature stability, and precise temperature control, with their performance largely dependent on the efficiency of the materials used. 

The energy conversion efficiency of a TE material is governed by a dimensionless parameter called the figure of merit *ZT* = (*S*^2^*σ*/*k*) *T*, where *S* is the Seebeck coefficient, *σ* is the electrical conductivity, and *k* is the thermal conductivity at a given temperature *T*, respectively [7]. In this way, by analyzing the equation, we can infer that a good candidate for a TE material presents a high *ZT* value, that is, it should have high *σ*, low *k*, and a high *S*. Moreover, the power factor parameter, *PF* = *S*^2^*σ*, is another way to measure the TE efficiency, which is directly proportional to the *ZT*. An increase in the *PF* value and a decrease in *k* leads to an increase in *ZT*.

Bi_2_Te_3_- and Sb_2_Te_3_-based alloys remain the most promising TE materials for near room-temperature applications due to their high conversion efficiency, even though a wide range of innovative TE materials have been proposed and extensively researched. Recent research on these telluride alloys has focused on enhancing their TE performance through two primary approaches: (i) material structure modification by doping or element enrichment, and (ii) development of heterostructures. C. Sudarshan et al. [8] demonstrated that Te-rich Bi_2_Te_3_ films exhibit good performance with a *PF* value of 2.7 mWK^−2^m^−1^. Interestingly, G. Sadowski et al. [9] reported that the combination of sputtered Mg and Bi materials resulted in epitaxially Mg_3_Bi_2_ films with room-temperature *PF* values of 0.2 mWK^−2^m^−1^. For flexible electronics, the fabrication of Bi_2_Te_3_/SWCNT, Bi_2_Te_2.7_Se_0.3_/PVDF, and Bi_2_Te_3_@PEDOT hybrid structures have demonstrated intriguing and promising *PF* values of 0.52, 0.12 and 0.74 mWK^−2^m^−1^, respectively [10,11,12]. Furthermore, when it comes to alloy doping, a review performed by Y. Saberi et al. [13] evidenced that the doping of Bi_2_Te_3_ compound with metals/semiconductors (such as Se, Sb, Cu, Ge, Ag, Pt, Sr, and others) has been the most appealing method for researchers to effectively enhance TE performance of BiTe alloys. We also highlighted the work developed by Chen et al. [14], in 2023, which investigated the effect of Se doping in *n*-type BiTe films to optimize the charge carrier properties. First, Bi_2_Te_3_ and Se were deposited on glass substrates by sputtering and thermal evaporation, respectively. By heating the Se film between 275 and 325 °C, Se vaporizes and diffuses into the BiTe, forming a BiTeSe film. The doping amount was controlled by the thickness of the Se film. This process allowed for *PF* values of 1.5 mWK^−2^m^−1^ at room temperature, which is eight times higher than undoped BiTe films. Related to heterostructures, G. Wang et al. [15] reported the fabrication of Bi_2_Te_3_/W and Bi_2_Te_3_/Sb multilayer structures with *PF* values of 1.79 and 1.57 mWK^−2^m^−1^, respectively. Likewise, materials based on Sb_2_Te_3_ have also been extensively studied and show great advances. L.F. Argemi et al. [16] achieved a high *PF* value of 1.9 mWK^−2^m^−1^ for Ag_3.9_Sb_33.6_Te_62.5_, through Ag doping optimization. Recently, a significant increase in TE *PF* from 0.7 mWK^−2^m^−1^, for Sb_2_Te_3_, to 2.4 mWK^−2^m^−1^, for Ni-doped Sb_2_Te_3_, was obtained using a solid-state reaction method in its production [17]. Among the promising results achieved through doping processes, it is important to note that, over the long term, the amount of doping of external elements can induce structural stress [18,19], affecting the structure stability and movement of the carriers, potentially resulting in device failure.

In addition to Bi_2_Te_3_ and Sb_2_Te_3_ films, the chalcogenide films such as GeTe and SnTe have gained significant attention due to their promising TE performance. Recent advancements in thin-film fabrication techniques have further enhanced their TE efficiency, by optimizing the carrier concentration, reducing the lattice thermal conductivity, and improving the grain boundary engineering. GeTe is inherently a *p*-type material due to Ge vacancies, while SnTe suffers from excessive hole concentration caused by intrinsic Sn vacancies. Thus, in these materials, controlling the point defects is the most important for developing high-performance TE films and devices [20]. For example, T. Ishibe et al. [21] reported a high TE *PF* value of 1.2 mWK^−2^m^−1^ in epitaxial GeTe thin films fabricated at 250 °C. This was achieved with defect control using domain engineering and atomic volatilization, through optimizing substrate temperature. Additionally, S. Xu et al. [22] achieved excellent TE properties of SnTe films, with interface engineering. The fabrication of oriented nanopillar structures proved to be an effective method for enhancing carrier transport while scattering phonons. An impressive PF value of 1.98 mW K^−2^ m^−1^ was achieved.

E.M.F. Vieira et al. [23,24] had already demonstrated enhanced TE performance for Bi_2_Te_3_ and Sb_2_Te_3_ films through the co-evaporation of Bi with Te and Sb with Te elements, respectively.

In this work, the TE properties of Bi_2_Te_3_ and Sb_2_Te_3_ alloys fabricated via thermal co-evaporation were enhanced by combining commercial Bi_2_Te_3_ and Sb_2_Te_3_ alloys with their constituent elements (Bi, Sb, and Te). The thermal evaporation method offers several advantages over alternative fabrication techniques, including (i) scalability and reproducibility, as this method is suitable for large-scale production with consistent results, unlike solution mixing techniques; (ii) compatibility with various materials including metals, semiconductors, and some organic materials without significant degradation; (iii) simplicity and cost effectiveness compared with molecular beam epitaxy (MBE), atomic layer deposition (ALD) and chemical vapor deposition (CVD) methods; and (iv) faster deposition rates compared to sputtering, making it more efficient for certain applications. Different deposition rates and substrate temperatures were explored. Thermoelectric, structural, and morphological characterizations were conducted at room temperature. The obtained results exceed some reported values for materials produced through extra doping or complex manufacturing processes, emphasizing that enriching commercial alloys is an effective and promising approach to enhancing the thermoelectric properties of telluride thin films. Furthermore, a µ-TE device for localized heating was successfully fabricated using the optimized films.

## 2. Experimental

### 2.1. Thin Film Fabrication and Characterization

**Figure 1:**
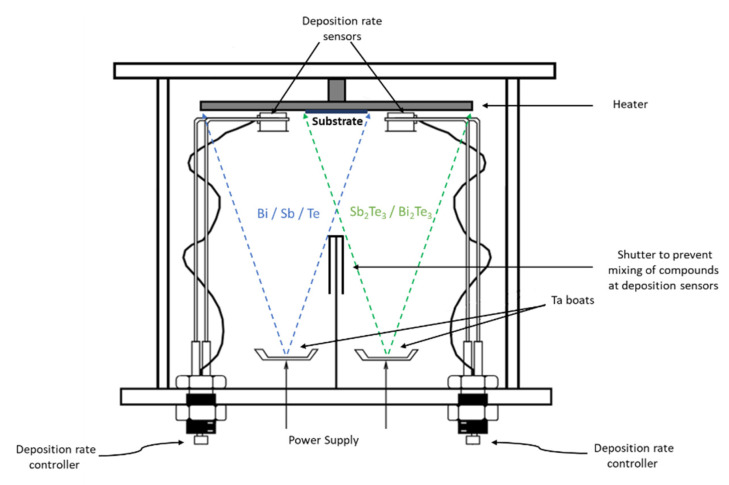
Vacuum chamber for co-evaporation of the different alloy combinations.

Thermal co-evaporated Bi_2_Te_3_ and Sb_2_Te_3_ thin films, 350 nm thick, were fabricated through commercially available Bi_2_Te_3_ (99.999% purity, CERAC^TM^ Incorporated; Milwaukee, WI, United States) and Sb_2_Te_3_ pieces (99% purity; CERAC^TM^ Incorporated; Milwaukee, WI, United States) combined with Bi pellets (99.999% purity; Kurt Lesker; Dresden, Germany), Sb pellets (99.999% purity; Kurt Lesker; Dresden, Germany) and Te lump (99.999% purity; Alfa Aesar; Waltham, MA, United States). Each material was evaporated from independent tantalum (Ta) boats, resulting in four alloy combinations, namely, (i) Bi_2_Te_3_:Bi; (ii) Bi_2_Te_3_:Te; (iii) Sb_2_Te_3_:Sb, and (iv) Sb_2_Te_3_:Te, as represented in Figure 1. Each evaporation boat was filled with the respective material to have a homogeneous evaporation. Prior depositions, borosilicate glasses (750 µm thick; MicroChemicals GmbH; Ulm, Germany) were cleaned with isopropanol, dried using an N_2_ flow, and positioned on a resistive heater to be used as substrates.

This work aims to investigate how enriching telluride alloys with their constituent elements (Bi; Sb or Te) affect their TE properties. The Bi_2_Te_3_ (or Sb_2_Te_3_) deposition rates were fixed at 3 Å/s, while the Bi (Sb or Te) deposition rates were varied between 1 and 4 Å/s. Each deposition rate was measured independently, with a crystal oscillator for each Ta boat. Moreover, the films were deposited at different substrate temperatures of 150, 250, and 300 °C.

The TE properties were investigated through in-plane Seebeck coefficient (*S*) and electrical conductivity (σ) measurements at room temperature (25 °C). The Seebeck coefficient (*S* = Δ*V*/Δ*T*) was measured using a custom-built setup based on the “two-probe” method [25]. Each film was positioned between two metal blocks: one maintained at room temperature, and the other heated to a fixed temperature by applying a series of voltage values (between 1 and 3 V) using a DC programmable source (Yokogawa model 7651; Yokogawa Electric Corporation, Tokyo, Japan). In this way, a temperature gradient of a few degrees Celsius across the film was created. The temperatures were monitored using *PT-100* sensors embedded in the metal blocks. The resulting thermo-voltage was recorded with an Agilent 34410A 6½ Digit Multimeter (Agilent Technologies Inc., Santa Clara, CA United States). *S* values, along with their associated errors, were calculated using the *LINEST* function in Microsoft Excel. Before measurements, the Seebeck setup was calibrated using a standard pure copper metal. A standard four-probe van der Pauw method was used for σ measurements. The values were determined by averaging three measurements taken at different points on the films. The means are presented along with their standard errors. The thermoelectric efficiency of the films was evaluated by the *PF* parameter, as referred to in the Introduction section.

The thickness, surface morphology, and chemical composition of the films were investigated by scanning electron microscopy (SEM) with integrated energy-dispersive X-ray spectroscopy (EDS) by using the JEOL JSM 6010LV equipment (JEOL Ltd., Tokyo, Japan). A 3 nm thick Pt film was coated on each investigated sample to improve SEM image quality.

The crystallographic structure was identified by X-ray diffraction (XRD) measurements, through the SmartLab Rigaku^®^ diffractometer. The X-ray diffraction (XRD) was carried out at room temperature over the range 2θ = 10–80° in a Bragg–Brentano θ/2θ configuration using Cu Kα radiation and λ = 1.540593 Å. The XRD operates with 9 kW power, 45 kV, and 200 mA.

### 2.2. Device Fabrication and Characterization

**Figure 2:**
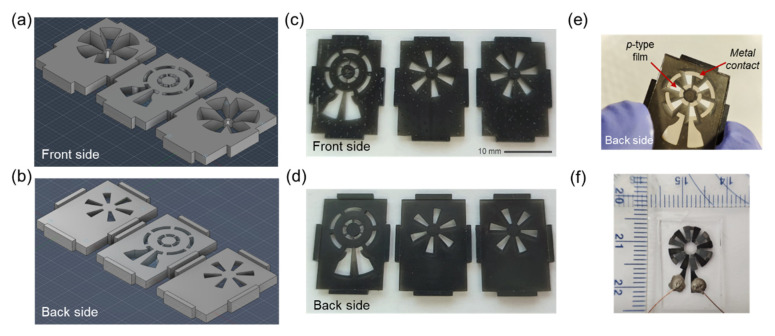
Three-dimensional model of the shadow masks with developed resin masks: front side (**a**,**c**) and backside (**b**,**d**). The back side is the side facing the substrate and the front side is the side that receives the material evaporation. The resin mask corresponding to the metal contacts is on the left side, followed by the mask corresponding to the *p*-type (center) and *n*-type (right side) materials. A photograph of the device to be built with two completed steps is shown in (**e**) and the final device in (**f**).

A ring-shaped thermoelectric device based on 6 *p-n* junctions of optimized *n-*Bi_2_Te_3_ and *p-*Sb_2_Te_3_ films was designed and fabricated by using 3D resin masks developed by the ANYCUBIC PHOTO MONO 4K printer. Figure 2a,b contains the 3D model of the shadow masks, designed in Autodesk Fusion 360 software. The photos of the printed parts are represented in Figure 2c,d. The resin masks have 14 × 20 mm (width × length) dimensions and contain supports on the 4 sides. Side supports serve two purposes: (i) to secure the glass substrates of identical size, and (ii) to ensure alignment between depositions, as the masks share the same dimensions. Additionally, the masks were designed with a chamfer to prevent the walls of one structure from obstructing the material deposition on adjacent structures. This manufacturing method for the masks was crucial, as it allowed for the production of parts with high dimensional precision. This precision was achieved thanks to the 4K LCD screen of the printing equipment, which provides an X and Y resolution of 34 μm (pixel size) and a vertical axis resolution of 10 μm.

By using these masks, we first deposited the metal contacts (1 µm of Al followed by 20 nm of Cr) by e-beam deposition on the glass substrate (V = 7kV; I = 180 mA), and then the Sb_2_Te_3_ legs followed by the Bi_2_Te_3_ legs, both 350 nm thick, using the optimized deposition parameters. The partially assembled device after two completed steps is shown in Figure 2e, while the final device is depicted in Figure 2f. The inner and outer diameters of the structure are 3 and 10 mm, respectively.

The total angle of each pair is 48.8°, the spacing of adjacent pairs is 1.2°, and the spacing with *n*/*p*-legs is 11.2°. The total area of the device is 71.31 mm^2^.

A maximum current of 4.8 mA was supplied to the device and the temperatures of the cold and hot side junctions were monitored using an infrared camera (Optris PI 450, Optris Infrared Sensing LLC., Portsmouth, NH, United States) placed 10 cm from the device.

## 3. Results and Discussion

### 3.1. Bi_2_Te_3_ and Sb_2_Te_3_ Alloys

**Figure 3:**
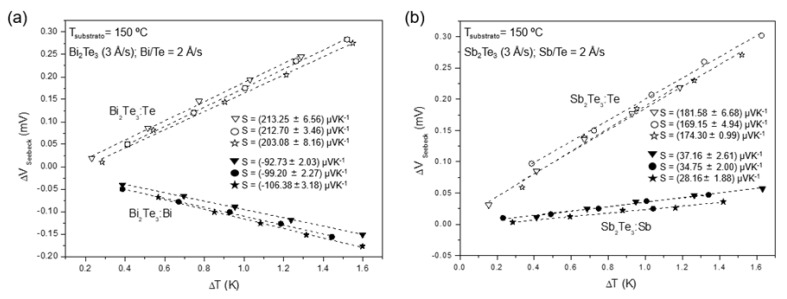
Seebeck voltage (Δ*V_Seebeck_*) vs. temperature gradient (Δ*T*) for (**a**) Bi_2_Te_3_:Te and Bi_2_Te_3_:Bi and (**b**) Sb_2_Te_3_:Te and Sb_2_Te_3_:Sb thin films, fabricated at 150 °C substrate temperature. Linear fits are added. Seebeck values and corresponding errors were added.

The thermo-voltage responses of Bi_2_Te_3_ and Sb_2_Te_3_ alloys fabricated at 150 °C and deposition rates of 3 Å/s (for Bi_2_Te_3_ or Sb_2_Te_3_) and 2 Å/s (for Bi, Sb or Te), are shown in Figure 3a,b. The depositions were performed three times for each alloy combination to assess reproducibility. As can be seen in Figure 3a, positive *S* values of (203.08 ± 8.16), (212.70 ± 3.46) and (213.25 ± 6.56) µVK^−1^ were measured for the Bi_2_Te_3_:Te alloys and negative values of (−92.73 ± 2.03), (−99.20 ± 2.27) and (−106.38 ± 3.18) µVK^−1^ were registered for the Bi_2_Te_3_:Bi alloys, indicating the formation of *p*-type Bi_2_Te_3_:Te and *n*-type Bi_2_Te_3_:Bi thin films. For Sb_2_Te_3_ alloys, depicted in Figure 3b, only positive S values were obtained, which were (37.16 ± 2.61), (34.75 ± 2.00), and (28.16 ± 1.88) µVK^−1^ for the Sb_2_Te_3_:Sb alloys and (181.58 ± 6.68), (174.30 ± 0.99), and (169.15 ± 4.94) µVK^−1^ for the Sb_2_Te_3_:Te alloys.

Moreover, by using the four-probe technique, *σ* values of (4.08 × 10^3^ ± 3.00), (3.99 × 10^3^ ± 2.40), and (3.93 × 10^3^ ± 1.90) (Ω m)^−1^ were obtained for *p*-type Bi_2_Te_3_:Te films, and (3.99 × 10^4^ ± 2.67 × 10^3^), (2.27 × 10^4^ ± 1.14 × 10^2^), and (1.59 × 10^4^ ± 2.2 × 10^1^) (Ω m)^−1^ were observed for *n*-type Bi_2_Te_3_:Bi films. *PF* values of (0.17 ± 0.01), (0.18 ± 0.01), and (0.18 ± 0.01) mWK^−2^m^−1^ were observed for Bi_2_Te_3_:Te films, while values of (0.34 ± 0.01), (0.22 ± 0.01), and (0.18 ± 0.01) mWK^−2^m^−1^ were observed for Bi_2_Te_3_:Bi films. Furthermore, higher *PF* values were obtained for Sb_2_Te_3_:Te films, namely (0.36 ± 0.03), (0.40 ± 0.01), and (0.43 ± 0.03) mWK^−2^m^−1^ when compared with Sb_2_Te_3_:Sb films ((0.14 ± 0.02), (0.15± 0.02), and (0.10 ± 0.01) mWK^−2^m^−1^). The corresponding electrical conductivities are (1.10 × 10^4^ ± 10.10), (1.32 × 10^4^ ± 9.51), and (1.52 × 10^4^ ± 8.4) (Ω m)^−1^ for Sb_2_Te_3_:Te films and (1.01 × 10^5^ ± 1325.2), (1.23 × 10^5^ ± 925.3), and (1.26 × 10^5^ ± 1247.3) (Ω m)^−1^ for Sb_2_Te_3_:Sb films, respectively.

Bi_2_Te_3_ is a good thermoelectric material for room-temperature applications, and its properties can be tuned to *p-* or *n-*type with corresponding element additions (Bi or Te). The maximum TE efficiency of a material is determined by its *PF* or *ZT*, while the maximum achievable *ZT* is defined by its quality factor, *B*. The quality factor *B* is associated with the weighted mobility µ_w_, which characterizes the relationship between the Seebeck coefficient and electrical conductivity, and with the lattice thermal conductivity, k_L,_ which quantifies heat transport by phonons [26]. The optimal type of Bi_2_Te_3_ film was selected based on the *B* values reported in the work of I.T. Witting et al. [26]. At room temperature, *n*-type Bi_2_Te_3_ exhibits a higher *B* value than *p*-type Bi_2_Te_3_, indicating a greater potential for achieving superior *PF* and *ZT*. Therefore, the following *PF* studies were conducted for *n*-type Bi_2_Te_3_. Considering the results for Sb_2_Te_3_ alloys, we can conclude that the TE properties are significantly enhanced when we have Te-rich SbTe films, thus revealing a tendency to reach the Te properties (*S* = 500 µVK^−1^ and σ = 1 × 10^4^ (Ω m)^−1^).

For these reasons, and to achieve high-quality bismuth and antimony telluride thin films, we further explored the Bi_2_Te_3_:Bi and Sb_2_Te_3_:Te alloy combinations by varying the substrate temperature and deposition rate.

### 3.2. Bi_2_Te_3_:Bi and Sb_2_Te_3_:Te Films: Substrate Temperature and Deposition Rate Variations

**Figure 4:**
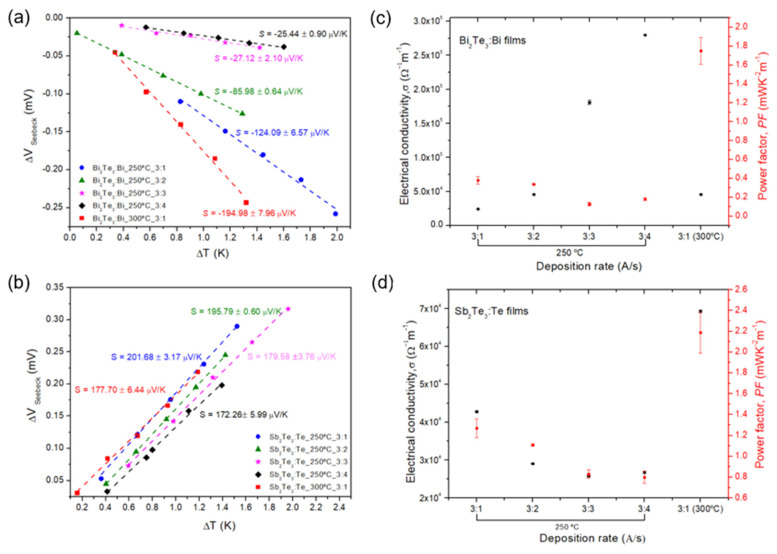
(**a**,**b**) *S* vs. Δ*T* for Bi_2_Te_3_:Bi and Sb_2_Te_3_:Te films fabricated at different substrate temperatures (250 and 300 °C) and deposition rates (3 Å/s (Bi_2_Te_3_):Bi, respectively). Linear fits are added for each alloy; (**c**,**d**) σ for Bi_2_Te_3_:Bi and Sb_2_Te_3_:Te films as a function of deposition rate, respectively. The standard deviation for each data point is represented by error bars, though some may be difficult to discern due to the scale.

In this section, different Bi_2_Te_3_:Bi and SbTe:Te films were fabricated at different substrate temperatures (250 and 300 °C) and deposition rates (being 3 Å/s for Bi_2_Te_3_/Sb_2_Te_3_ and 1,2,3, and 4 Å/s for Bi/Te). As observed in Figure 4a,b, the measured *S* values indicate that all Bi_2_Te_3_:Bi and Sb_2_Te_3_:Te films have *n*-type and *p*-type conductivities, respectively. When the temperature increases from 150 to 250 °C, at a deposition ratio of 3:2 Å/s, a small decrease in *S* value is observed for the Bi_2_Te_3_:Bi film when compared with the film fabricated at 150 °C (see Section 3.1). However, the observed increase in the σ value results in a higher *PF* value of (0.34 ± 0.01) mWK^−2^m^−1^ (Figure 4c). Moreover, for a substrate temperature of 250 °C, Figure 4a, it is clear that *S* remarkably decreases with the increasing of Bi content in the Bi_2_Te_3_ alloy and, in turn, σ increases. The notable difference of almost one order of magnitude in σ (from ~10^4^ to ~10^5^) is attributed to the enrichment of Bi content in the alloy, with σ values approaching those of the pure Bi structure (σ (Bi) = 7.7 × 10^5^ (Ω m)^−1^) [27]. *S* results further support this observation, as they closely align with *S* values for Bi structure [28,29]. A maximum *S* of −124.09 ± 6.57 µVK^−1^ was obtained for the Bi_2_Te_3_:Bi film, fabricated at 250 °C with a deposition rate of 3:1 Å/s. Interestingly, when the substrate temperature increases to 300 °C, a remarkable increase in the *S* value is observed (from −124 to −195 µVK^−1^), which results in an excellent *PF* value of 1.75 mWK^−2^m^−1^.

A similar procedure to the one used for optimizing Bi_2_Te_3_ films was adopted for Sb_2_Te_3_:Te alloys. The rise in the *PF* value (*PF* = (1.11 ± 0.01) mWK^−2^m^−1^) when the substrate temperature increases from 150 °C to 250 °C, for the Sb_2_Te_3_:Te film fabricated at 3:2 of deposition rate, respectively, is primarily attributed to the increase in *S*. This result is observed in Figure 4d. However, considering the films fabricated at 250 °C of substrate temperature, a slight decrease in *S* is observed as the Sb_2_Te_3_ alloy becomes more enriched with Te, unlike what happens in Bi_2_Te_3_:Bi films. Furthermore, when we compare the SbTe:Te films fabricated at 250 and 300 °C (at 3:1 of deposition rate, respectively), it seems that the re-evaporation effect of Te from the substrate is much more important when the temperature is 300 °C, due to the significant decrease in *S*, accompanied by an increase in σ, resulting in a *PF* of (2.19 ± 0.20) mWK^−2^m^−1^.

**Figure 5:**
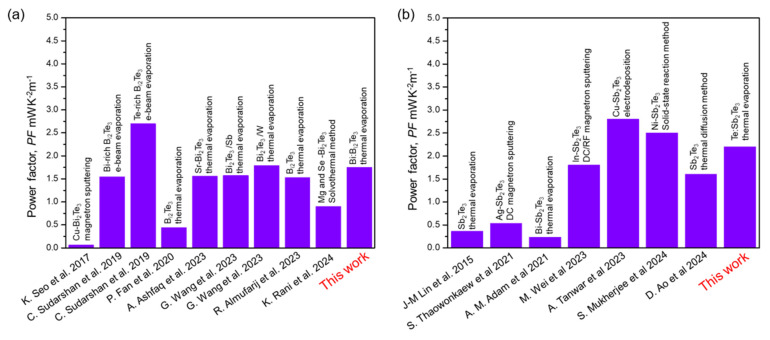
Comparison of the *PF* value of several reported: (**a**) Bi_2_Te_3_- and (**b**) Sb_2_Te_3_-based alloys, fabricated with different techniques [8,15,17,30,31,32,33,34,35,36,37,38,39,40]. Our values are comparable to or greater than those obtained in reference works. [8,15,17,30,31,32,33,34,35,36,37,38,39,40].

Our *PF* values are comparable to or greater than those obtained in reference works [8,15,17,30,31,32,33,34,35,36,37,38,39,40], some of which incorporate additional elements into the system, unlike the work presented here (Figure 5). C. Sudarshan et al. [8] reported e-beam-evaporated Te-rich Bi_2_Te_3_ with higher *PF* than our *PF* value. However, we highlight that our fabrication method is more simplistic since no additional annealing procedure is required. All fabrication steps are performed in the vacuum chamber. Considering Sb_2_Te_3_ alloys, A. Tanwar et al. [35] reported higher *PF* for CuSbTe compounds by the electrodeposition method. Nevertheless, limited scalability, high costs, and non-uniform results are drawbacks to the reproducible production of these materials. Moreover, enhanced *PF* Sb_2_Te_3_ materials were synthesized due to Ni doping by the solid-state reaction method [17]. Although it is a simple process that does not require expensive equipment, some inherent disadvantages of this process include the need for high temperatures, the possibility of non-homogeneity, and, in some cases, uncompleted reactions.

**Figure 6:**
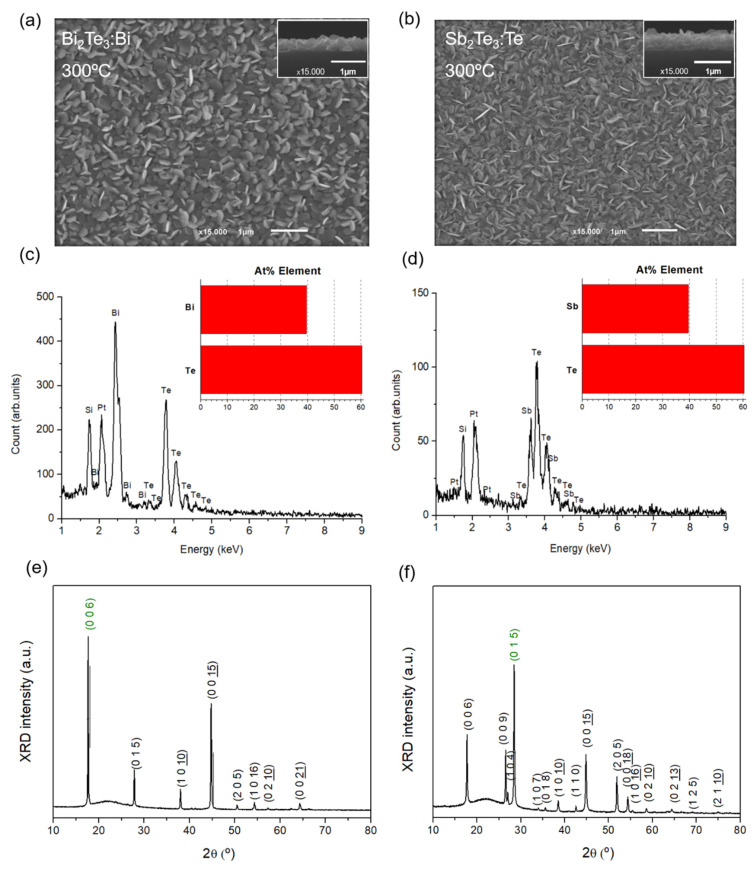
Morphology and structural properties of Bi_2_Te_3_:Bi and Sb_2_Te_3_:Te films fabricated at 300 °C: (**a**,**b**) surface SEM image acquired at the center of the film (inset: cross-section analysis), (**c**,**d**) EDS data, and (**e**,**f**) XRD spectrum showing stoichiometric rhombohedral Bi_2_Te_3_ and Sb_2_Te_3_ films.

To complement the thermoelectric properties, SEM, EDS, and XRD measurements were conducted to investigate the morphology and structural characteristics of the films that exhibited the highest *PF* value. The Bi_2_Te_3_:Bi and Sb_2_Te_3_:Te films fabricated at 300 °C reveal a lamellar flake morphology, as evidenced in Figure 6a,b, without visible defects and/or microcracks, resulting in dense and compact structures. The thicknesses of the films were estimated from the cross-sectional SEM measurements and were found to be 350 ± 10 nm. The EDS spectra, represented in Figure 6c,d, also reveal that both films are Te-rich, with atomic% ratio between Te and Bi (or Sb) elements of 1.5 (~40% Bi (Sb) and ~60% Te), corresponding to stoichiometric Bi_2_Te_3_ and Sb_2_Te_3_ films. The observed Si and Pt peaks are attributed to the glass substrate and the coating, respectively, as mentioned in the experimental section.

Likewise, as can be seen in Figure 6e,f, all the diffraction peaks are indexed to the rhombohedral phases of Bi_2_Te_3_ (JCPDS card no. 15-0863) and Sb_2_Te_3_ (JCPDS card no. 71-0393). No impurities peaks are observed. For the Bi_2_Te_3_:Bi film, the (0 0 6) and (0 0 15) peaks are the two main peaks, but the strongest peak is related to the (0 0 6) orientation plane, indicating (00*l*) preferred (*c*-axis) orientation of the film fabricated at 300 °C. Information on crystallite size for the films was calculated through the Debye Scherrer formula [41] using the two main orientation planes of the XRD spectrum of the Bi_2_Te_3_:Bi film, which corresponds to 49.1 nm. For the Sb_2_Te_3_:Te film, the highest peak is (0 1 5) indicating preferred crystallite growth orientation in the (0 1 5) plane. The nanoparticle size was found to be 45.9 nm.

The Seebeck coefficient (S) and electrical conductivity (σ) of a material are strongly influenced by deposition rate and temperature. The deposition rate influences the nucleation and growth dynamics of the material [42]. A low deposition rate allows enhanced atomic mobility and grain growth, leading to larger grains with fewer boundaries. Moreover, this parameter affects the alloy composition. According to previous work [23], films with higher content of Te than Bi (or Sb) show higher *S* values and lower *σ* values when compared with Bi or Sb-rich films. On the other hand, the deposition temperature governs atomic mobility, crystallinity, and phase formation in the material [43]. High temperature promotes better crystallization and leads to improved order and fewer defects. A dense, well-ordered, and crystalline structure is expected to be beneficial for carrier’s transport and phonon scattering. In this way, an optimization of *S* and *σ* is expected. In conclusion, the *n*-type Bi_2_Te_3_ and *p*-type Sb_2_Te_3_ films fabricated at 3 Å/s (for Bi_2_Te_3_ and Sb_2_Te_3_) and 1 Å/s (for Bi and Te), combined with 300 °C of substrate temperature, are suitable for the development of a high-performance TE device.

### 3.3. Thermoelectric Device

Based on the thorough and detailed characterization study, we infer that the developed TE thin films are well-suited for thermal sensor development. As a proof-of-concept, a *p-n* TE device has been successfully designed and fabricated for localized heating applications. As described in Section 2.2, approximately 1 μm of Al and 20 nm of Cr were initially deposited using the e-beam deposition method to produce the metal contact between the *p*-type and *n*-type legs of the device, as represented in Figure 2e. Then, six *n*-type and six *p*-type legs were deposited by thermal co-evaporation, using the previously optimized conditions (thickness = 350 nm; deposition rate = 3 Å/s (for Bi_2_Te_3_ and Sb_2_Te_3_) and 1 Å/s (for Bi and Te; substrate temperature = 300 °C). All depositions were performed with the resin masks of Figure 2c,d, placed on a 750 µm thick borosilicate glass substrate, to ensure that the patterns of each material are aligned correctly. Seebeck coefficients of −195 and 178 µVK^−1^, and electrical resistivity of 22 and 14 µΩ.m were measured for the optimized *n*-type and *p*-type films, respectively, corresponding to the highest *PF* values of 1.75 and 2.19 mWK^−2^m^−1^.

**Figure 7:**
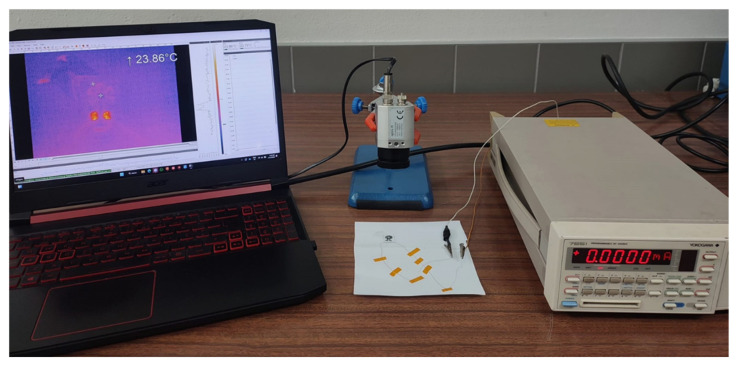
Setup for thermal characterization of the developed TE device.

After the device fabrication, two copper wires were attached to its external contacts with liquid silver paint to improve electrical conductivity, as evident in Figure 7. The copper wires were then connected to the YOKOGAWA 7651 power supply (Yokogawa Electric Corporation, Tokyo, Japan) to serve as the current source of the device, and the performance of the device (temperature gradient formed between the cold and hot sides) was recorded using the Optris PI thermal camera at a distance of 10 cm from the device in atmospheric conditions and without any heat sink attached to the device. Before the measurements, wire thermocouples were used to measure the surface temperature for calibration. The difference between the temperatures obtained from the thermocouples and those from the thermal camera was found to be less than 1%, as already observed in our last work [24].

**Figure 8:**
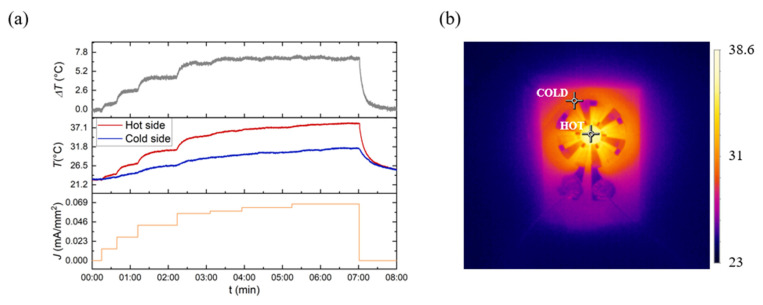
(**a**) Real-time temperature measurements at different applied currents. A temperature difference of up to 7 °C is observed at the current of 4.8 mA (*J* = 0.068 mA/mm^2^). (**b**) Typical thermal image of the developed device captured using an infrared (IR) camera when the device is supplied with 4.8 mA of current.

Figure 8a contains real-time temperature measurements under different biased currents, conducted to demonstrate the device’s capability of generating a temperature variation (Δ*T*). The data were gathered using the thermal camera software. Firstly, we can identify the points where the current was changed, as the test was performed with fixed current values steps rather than a gradual increase. These steps are shown in the graph, beginning with the current at 0 mA and ending at 4.8 mA (*J* = 0.068 mA/mm^2^). After the 4 mA current mark, the steps do not maintain the same increase. This is because as the current was changed from 4 mA to 5 mA, the device would require more power than it could receive from the current source, so the steps in the current needed to be less than the previous steps. It is possible to conclude that as the current increases, the temperature of the hot and cold zones rises simultaneously, quickly reaching a saturated Δ*T*. The thermal energy within the TE heat/cool device is influenced by several factors, namely the Peltier effect and the thermal conduction energy from the Joule heat generated by the internal resistance of the device. Although there is a contribution from both effects, the Joule effect overrides the Peltier effect, causing the temperature rise of the cold zone, and therefore, a saturation point at the Δ*T* value is achieved. However, a high temperature of 38.6 °C was recorded on the hot side, while a maximum temperature of 31.5 °C was reached on the cold side, resulting in a maximum temperature differential of 7.1 °C. Our TE device shows higher Δ*T* values than the ones revealed by TE devices with similar TE material, higher TE material thicknesses (in the order of micrometers), and higher applied current [44,45,46,47].

The corresponding thermal image of the device supplied by 4.8 mA of current, with a color scale matching the various temperatures detected by the camera, was depicted in Figure 8b. The hot zone (center of the device) and cold zone (periphery of the device) are easily identified. However, the thermal contact between the TE materials and the metal contacts appears to be poor, as indicated by the substantial thermal gradient observed in this region. These results could be attributed to the high contact resistance observed. Further work will focus on decreasing the internal resistance by changing the metal contacts and/or optimizing the device geometry design (thickness, number, and/or length/weight ratio of legs).

## 4. Conclusions

This study demonstrates the enhancement of TE properties of Bi_2_Te_3_ and Sb_2_Te_3_ thin film alloys through thermal co-evaporation with their constituent elements (Bi, Sb, and Te). By optimizing deposition parameters, such as substrate temperature and deposition rates, the fabricated thin films (350 nm thick) exhibit *PF* values of 1.75 mW K^−2^ m^−1^ for *n*-type Bi_2_Te_3_ and 2.19 mW K^−2^ m^−1^ for *p*-type Sb_2_Te_3_ at 300 °C. These *PF* values surpass several previously reported results, emphasizing the efficiency of this approach without the complexity of strategies like external doping or post-deposition treatments. The structural and morphological analyses confirmed stoichiometric compositions, with preferred crystalline orientations, compact structures, and minimal defects.

Additionally, the successful fabrication and testing of a ring-shaped thermoelectric device with six *p-n* junctions further validates the practical potential of these optimized films for localized heating applications. A maximum temperature gradient of 7.1 °C was achieved using 4.8 mA of current, demonstrating the device’s efficiency and reliability. The results and observations gathered throughout this work highlight the potential of enriching commercial TE alloys with constituent elements as a scalable, cost-effective, and straightforward strategy to improve their TE properties. This makes it suitable for developing high-performance TE materials for energy harvesting, thermal sensors, and micro-scale heating applications.

## Data Availability

Data is contained within the article.
